# LLM-in-the-Loop execution of clinical quality language

**DOI:** 10.1093/jamiaopen/ooag099

**Published:** 2026-06-13

**Authors:** Bell Raj Eapen, Oladimeji M Adaramewa, Xiaoqing Li

**Affiliations:** Management Information Systems, University of Illinois, Springfield, IL, United States; Management Information Systems, University of Illinois, Springfield, IL, United States; Management Information Systems, University of Illinois, Springfield, IL, United States

**Keywords:** Clinical quality language, large language models, clinical decision support, unstructured text, FHIR

## Abstract

**Objective:**

We present a method and open-source prototype to augment Clinical Quality Language (CQL) using a large language model (LLM) in the execution loop for querying unstructured text.

**Materials and Methods:**

We modified a popular CQL engine to trigger an LLM-in-the-Loop pipeline (LitL) whenever CQL references unstructured FHIR resources, without altering the existing structured resource handling. LitL generates a binary response to natural language queries from CQL using an LLM.

**Results:**

We present an open-source prototype that includes: an implementation of the LitL pattern, and a modified CQL execution engine that triggers LitL for unstructured data. The feasibility testing with two locally hosted LLMs achieved an accuracy of 72% and 93% respectively. An end-to-end prototype is provided to facilitate implementation with configurable models, prompts and hyperparameters.

**Conclusion:**

The LitL pattern extends CQL to unstructured data, preserving compatibility with existing CQL and reducing hallucination. All components of the prototype are free and open source.

## Introduction

### Background and significance

CQL^1^ is a domain-specific language developed to standardize the expression of clinical logic across healthcare applications, particularly in electronic clinical quality measures (eCQMs) and clinical decision support systems (CDSS).^1^ It allows clinicians and researchers to write queries and retrieve data from electronic health records (EHRs) in a standardized and interoperable way. For example, CQL can be used to check whether a patient has a diagnosis code for diabetes, a medication list that includes insulin, or a lab value such as HbA1c, all of which are examples of structured data stored in coded formats. It is important to distinguish CQL from Cassandra Query Language (also CQL), which is a database query language, and Structured Query Language (SQL). While SQL and Cassandra CQL are designed for database retrieval, Clinical Quality Language is designed for expressing clinical logic and reasoning over healthcare data models like FHIR (Fast Healthcare Interoperability Resources). FHIR is a widely adopted standard for exchanging healthcare information electronically, defining resources such as Patient, Condition, and Observation.^2^

CQL expressions are compiled to Expression Logical Model (ELM) in JSON or XML for execution at runtime, enabling interoperable, machine-readable logic that can be consistently interpreted across diverse health IT systems.^3^ However, CQL is currently limited to structured data sources, such as coded diagnoses and medication lists. This constraint poses a significant challenge in modern clinical environments, where a substantial portion of valuable patient information exists in unstructured formats such as free-text clinical notes, radiology and pathology reports, among others. For example, determining if a patient has “documented signs of infection” often requires analyzing free-text notes, which standard CQL cannot do. Tools like SmartChartSuite^4^ have attempted to address similar challenges in data enrichment, but seamless integration into the CQL execution flow remains a gap.

The inability of CQL to natively query or interpret unstructured text restricts its effectiveness in capturing the full clinical context. Recent advances in natural language processing (NLP), particularly through LLMs, enable scalable interpretation of unstructured text.^5^

## Objective

Our objective is to enhance the expressiveness and utility of CQL by introducing support for unstructured text queries, while preserving the standard CQL workflow for structured data. CQL, as a standardized language for clinical logic representation, currently operates on structured data, limiting its applicability in scenarios where clinical information is embedded in a free-text format in clinical notes, discharge summaries, or lab reports.^6^ To address this limitation, we propose a novel framework that integrates large language models (LLMs) into the CQL execution pipeline, enabling the querying of unstructured clinical text. Additionally, we propose a method for embedding unstructured resources within CQL expressions. We prototyped the necessary tools and demonstrated the feasibility of querying unstructured text using CQL enhanced with LLMs. Finally, we propose an architecture that ensures interoperability and scalability of the enhanced CQL framework within existing health IT systems and standards. By bridging the gap between structured logic and unstructured clinical data, we aim to improve the precision and reach of CDSS, quality measurement, and health data analytics.

## Materials and methods

The proposed solution introduces a novel method for integrating LLMs into CQL execution engines to support unstructured data processing. This approach (LitL) selectively employs LLMs on unstructured FHIR resources such as “DocumentReference,” while structured resources like “Condition” are processed using traditional CQL logic.

### Architecture

**Figure 1.**
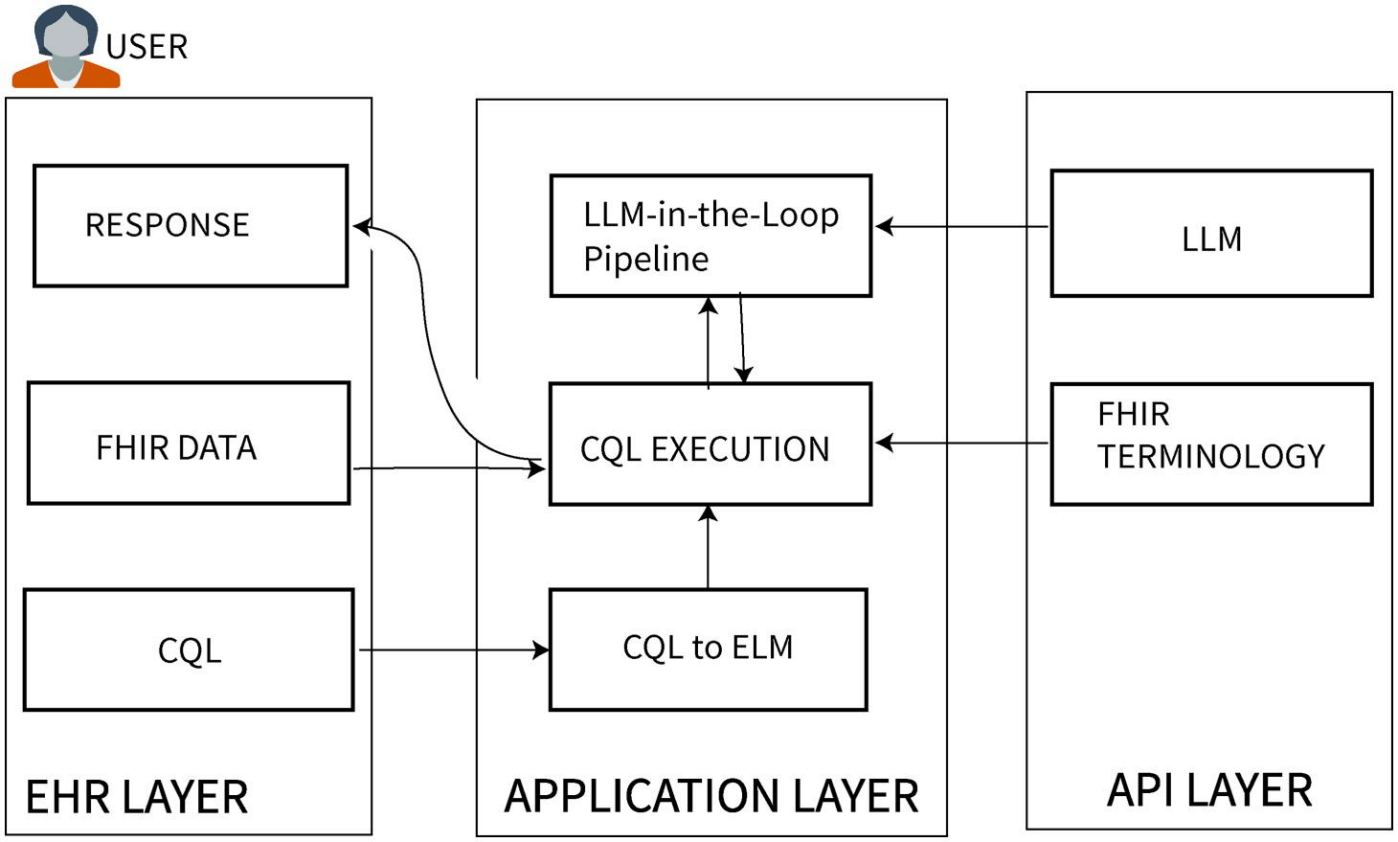
Three-layer system architecture for unstructured data processing in Clinical Quality Language (CQL). The architecture comprises: (1) the *EHR layer*, which provides source clinical data (FHIR) and displays response; (2) the *Application layer*, which manages unstructured data processing and workflow logic (expanded in Figure 2); and (3) the *API layer*, which enables standardized data exchange and integration.

The architecture comprises three layers (Figure 1): the application programming interface (API) layer, the application layer, and the EHR layer. The API layer provides self-hosted or external LLMs and a FHIR terminology service. We used locally hosted models and an external FHIR terminology server^7^ for testing. The application layer, implemented using the LangChain framework,^8^ orchestrates the interaction between CQL logic and the LLM, enabling dynamic interpretation of clinical notes. The EHR layer provides the user interface and data integration. Local LLM deployment keeps PHI on-premises, reducing exposure compared with remote API calls.

### System components

The system components include (Figure 1):

- FHIR data API and response interface^9^
- A CQL-to-ELM JSON converter.^10^
- A modified CQL execution engine that invokes the LitL pipeline when unstructured data reference is encountered.^11^
- LitL pipeline^12^ using LLM.
- FHIR Terminology API.

### LitL implementation

**Figure 2.**
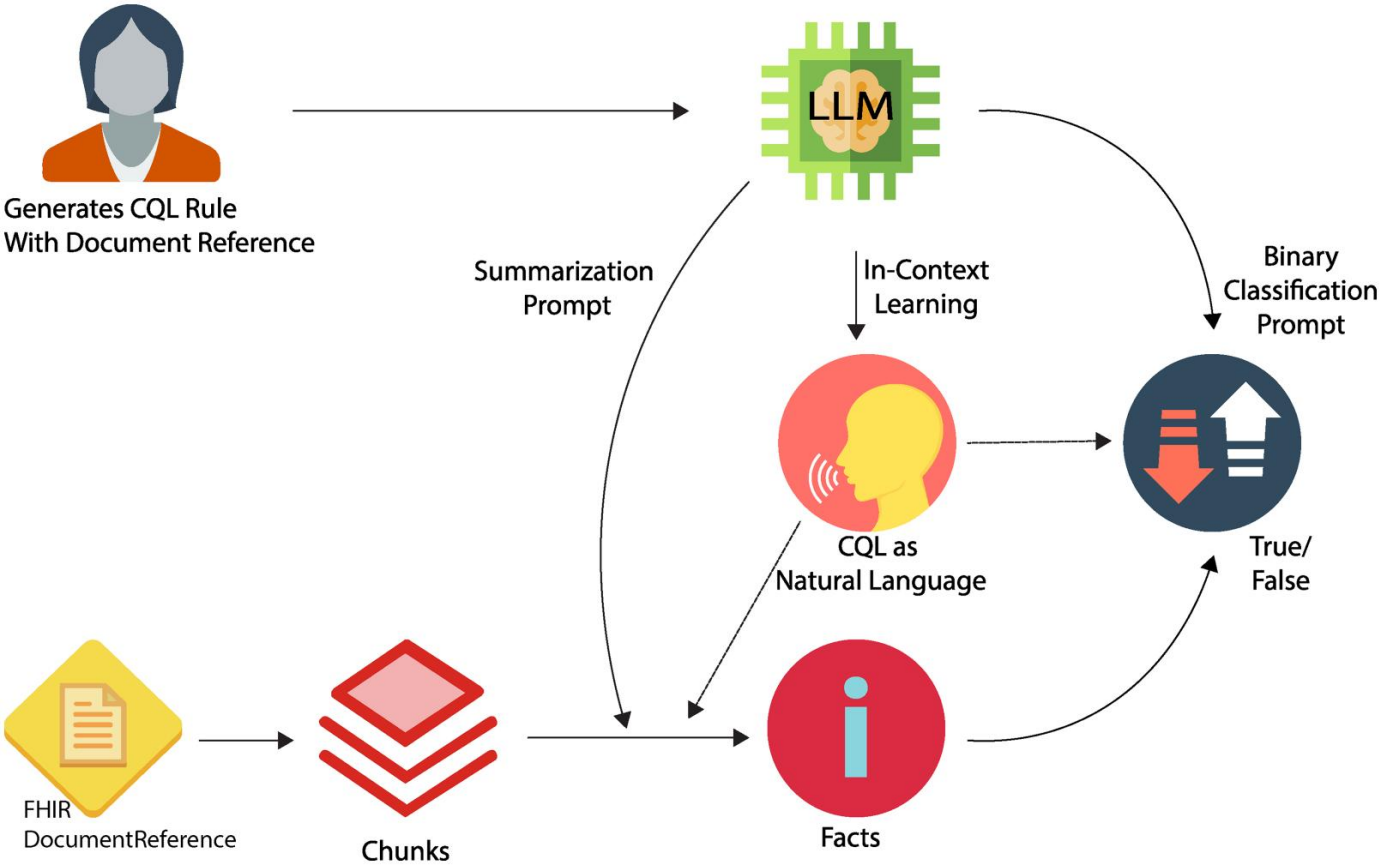
Data flow in the LitL pipeline for Clinical Quality Language (CQL) execution on unstructured data. The pipeline consists of: (1) document chunking of clinical notes retrieved from the EHR as DocumentReference FHIR resource, (2) mapping CQL expressions to natural language queries using in-context learning,(3) extracting query‑relevant facts from document chunks using summarization prompts with the translated natural‑language CQL query injected, and (4) generating a binary response (True/False) that is passed back into the CQL execution pipeline to complete the logic evaluation.

After converting CQL to ELM, the pipeline (see Figure 2) translates any CQL statement referencing unstructured data into a natural language query. This is accomplished through in-context learning, where the LLM is prompted with examples to generate a human-readable query. Next, unstructured clinical notes are segmented into chunks based on the LLM’s context window size. Each chunk is processed using a summarization prompt that includes the natural‑language query to extract query-relevant facts. These facts are then collated into a single document that can be evaluated against the natural language query. LLMs with large context windows can often process entire documents directly, eliminating the need for the chunking step. In the final step, the LLM evaluates the natural language query against the collated facts to produce a binary response using an appropriate classification prompt. This response is then used to determine whether the unstructured data satisfies the CQL statement.

The LitL pipeline is implemented using Python LangChain framework. We used a locally hosted LLM for testing the pipeline. The codebase of the system components is open-source and available on GitHub, encouraging community contributions.

Structured data continues to be processed using conventional methods, ensuring compatibility and minimizing disruption to existing workflows. To facilitate adoption, we provide an end-to-end implementation^9^ and instructions that helps implementers in setting up the stack and integrate it with EHR through FHIR interface.^13^

### Feasibility test

To evaluate the feasibility and performance of LitL, we conducted an experiment using a subset of clinical notes from the MIMIC-IV dataset.^19^ The goal was to assess the system’s ability to infer binary conditions from unstructured text in response to CQL queries. We manually crafted 100 CQL statements, 50 positives and 50 negatives, targeting clinical scenarios that referenced clinical notes. A “positive” CQL statement is one where the condition is expected to be met based on the text (e.g, “Documented diagnosis of diabetes” when the note says “Patient has type 2 diabetes”). A “negative” statement is one where the condition is not met (e.g, “Documented Myocardial infarction” when the note lacks that information). See Table 1 for hypothetical CQL referring unstructured FHIR resources and their natural language representation.

**Table 1.** Segments of CQL referring to an unstructured FHIR DocumentReference resource and its natural language representations.

CQL segment	Natural language query
…. exists ([DocumentReference] D where D.procedure=“Cardiac catheterization” and D.diagnosis=“Myocardial infarction” and D.finding=“ST elevations “) ….	Documented diagnosis of Myocardial infarction and cardiac catheterization procedure, with ST elevations finding.
…. exists ([DocumentReference] D where D.complaint=“Facial weakness” and D.pastMedicalHistory=“Hypertension” and D.familyHistory=“Stroke”) ….	Documented complaint of Facial weakness, past medical history of hypertension and family history of Stroke.

We tested the pipeline using two open-source models: Phi-3-Mini-4K-Instruct^14^ and Llama 3.1(8B)^15^ and the results are summarized in Table 2.

**Table 2.** Results of feasibility testing using two locally hosted LLMs.

Model	TP*	FP*	TN*	FN*	Accuracy	Precision	Recall	F1 Score
Phi3-Mini-4k-Instruct	39	17	33	11	0.72	0.70	0.78	0.74
Llama 3.1(8B)	46	3	47	4	0.93	0.94	0.92	0.93

* TP, True positive; FP, False positive; TN, True negative; FN, False negative.

## Results

The proposed architecture (Figure 1) augments CQL execution by adding support for unstructured FHIR resources.^11^ The LitL pipeline (Figure 2) implements document chunking, extraction of supporting facts and binary response for processing by the CQL engine.^12^ All system components are available as open-source and an end-to-end server application demonstrates their use.^9^

Results of the feasibility test are summarized in Table  2, with the larger model, Llama 3.1(8B), demonstrating higher accuracy, precision, and recall than Phi‑3‑Mini‑4k‑Instruct, driven primarily by a substantial reduction in false positives and a modest decrease in false negatives.

## Discussion

The LitL framework offers a practical and extensible solution to a longstanding limitation in clinical logic execution: the inability to process unstructured data. LitL framework enables the interpretation of free-text clinical documentation while preserving compatibility with existing structured data workflows.

The integration of LLMs into CQL execution engines has the potential to revolutionize clinical decision support and quality measurement. Though existing approaches such as cTAKES’s NegEx module^16^ can be used to extract negation context from clinical notes for CQL, they typically require custom dictionaries, and system maintenance.

Our feasibility test shows that the LitL can effectively extend CQL’s capabilities to unstructured data sources, addressing the limitation in the standard. The classification metrics vary depending on the models, prompts, and hyperparameters used. Previously reported classification accuracy of proprietary LLMs on discharge summaries ranges from 57% to 89%,^17^ while only entity extraction which is not directly comparable, has a high accuracy rate (89%-100%).^18^ We achieved 72% accuracy with a smaller model and 93% accuracy with the larger model. This suggests that even comparatively small, resource-efficient LLMs can contribute meaningfully to clinical logic execution when appropriately scoped and prompted. Such models may be sufficient for use cases including quality measurement queries (e.g, percentage of diabetic patients with HbA1c checked). LitL allows the use of any LLM, prompts and hyperparameters, all injected at runtime, according to the requirements of a given clinical domain.

LitL approach can extend CQL’s capabilities to unstructured data domains. While the feasibility test shows promise, domain-targeted evaluation is required to identify the best CQL, model, prompts and hyperparameters for each use case.

Compared with retrieval-augmented generation over notes or end-to-end generative extraction, LitL constrains the task to a binary decision grounded in CQL intent. By invoking the LLM pipeline only when unstructured data is referenced, we minimize computational overhead and reduce the risk of hallucinations, an important consideration in clinical contexts. This targeted approach also aligns with cost-efficiency goals, making the framework suitable for deployment in low-resource settings. The integration pattern ensures backward compatibility with existing CQL execution engines, preserving established workflows while enabling new capabilities. This design choice facilitates adoption by health IT vendors and institutions without requiring extensive reengineering of their systems.

Clinical decision support requires more rigorous error handling than clinical quality measurement, since it directly impacts patient safety. While our current implementation focuses on architectural feasibility, future iterations should incorporate error handling mechanisms to detect uncertain outputs and escalate them for human review. Additionally, the accuracy of LLM responses is sensitive to prompt design and model selection. LitL is model agnostic and more capable LLMs or distilled model variants may further improve accuracy and reduce latency. Further, the open-source availability makes the framework suitable for widespread adoption and collaborative improvement. Future work will focus on incorporating error handling mechanisms, refining the natural language query generation process, and conducting socio-technical evaluations in operational EHR environments.

## Conclusion

Overall, the LitL framework offers a practical and extensible solution for bridging the gap between structured logic and unstructured clinical data. Our preliminary tests show that even lightweight, locally hosted LLMs can provide meaningful support for unstructured data inferencing, achieving promising accuracy with minimal computational overhead. By enhancing the expressiveness of CQL, it opens new avenues for comprehensive, context-aware clinical decision support and quality measurement.

As healthcare data continues to grow in complexity and volume, tools like LitL will be essential for unlocking the full potential of clinical decision support and quality measurement. By bridging the gap between structured logic and unstructured clinical narratives, this work lays the foundation for more comprehensive, context-aware, and intelligent health IT systems.

## Data Availability

The source code of all the components of the LitL framework is available under a permissive open-source license from various GitHub repositories as listed in the references. Some of the repositories are forks of existing applications modified to implement additional features. The feasibility test was conducted using a subset of clinical notes from the MIMIC-IV dataset.^19^ No proprietary or patient-identifiable data were used in this study. An end-to-end application integrating all described components are available at https://github.com/dermatologist/cql-express-r4. Additionally, we have provided detailed step-by-step instructions for implementers to set up the framework on a public blog post.^13^
